# Impact of pre-diagnostic triglycerides and HDL-cholesterol on breast cancer recurrence and survival by breast cancer subtypes

**DOI:** 10.1186/s12885-018-4568-2

**Published:** 2018-06-15

**Authors:** Trygve Lofterød, Elin S. Mortensen, Hawa Nalwoga, Tom Wilsgaard, Hanne Frydenberg, Terje Risberg, Anne Elise Eggen, Anne McTiernan, Sura Aziz, Erik A. Wist, Andreas Stensvold, Jon B. Reitan, Lars A. Akslen, Inger Thune

**Affiliations:** 1 Department of Oncology, Oslo University Hospital, Ullevål, N-0424 Oslo, Norway; 2 Department of Clinical Pathology, University Hospital of North Norway, N-9019 Tromsø, Norway; 3 Centre for Cancer Biomarkers CCBIO, Department of Clinical Medicine, University of Bergen, N-5007 Bergen, Norway; 4 Department of Community Medicine, Faculty of Health Services, UIT The Arctic University of Norway, N-9019 Tromsø, Norway; 5 Department of Oncology, University Hospital of North Norway, N-9019 Tromsø, Norway; 6 Fred Hutchinson Cancer Research Center, Public Health Sciences Division, Seattle, WA 98109 USA; 7 Department of Oncology, Østfold Hospital Trust, N-1714 Grålum, Norway; 8 Department of Pathology, Haukeland University Hospital, N-9019 Bergen, Norway; 9 Institute of Clinical Medicine, Faculty of Health Services, UIT The Arctic University of Norway, N-9019 Tromsø, Norway

**Keywords:** Breast cancer, Lipids, Molecular subtype, Triple negative breast cancer, Survival

## Abstract

**Background:**

High triglycerides and low levels of high density lipoprotein (HDL)-cholesterol are observed to promote tumor growth. However, whether breast cancer heterogeneity may explain the contradictory influence of triglycerides and cholesterol observed on breast cancer prognosis remains unclear.

**Methods:**

A population-based survival study among 464 breast cancer cases identified within the Tromsø study was conducted. Pre-diagnostic triglycerides, total-cholesterol and HDL-cholesterol were measured, and detailed clinical and histopathological data were obtained. Using tissue microarray, all breast cancer cases were reclassified into the following subtypes: Luminal A, Luminal B, HER2-enriched, and triple negative breast cancer (TNBC). Multivariable Cox proportional hazards regression models were used to study the associations between pre-diagnostic lipids and breast cancer recurrence, mortality, and survival.

**Results:**

A total of 464 breast cancer patients, with mean age at diagnosis of 57.9 years, were followed for a mean 8.4 years. TNBC patients in the highest tertile of triglycerides (≥ 1.23 mmol/l) had 3 times higher overall mortality compared to TNBC patients in the lowest tertile (≤ 0.82 mmol/l) (HR 2.99, 95% CI 1.17–7.63), and the 5-year overall survival was 19% lower for TNBC patients in the highest vs. lowest tertile of triglycerides (65% vs. 84%). TNBC patients in the highest tertile of the HDL-cholesterol/total-cholesterol ratio (≥0.35), compared to those in the lowest tertile (≤0.27), had a 67% reduced overall mortality risk (HR 0.33, 95% CI 0.12–0.89). No associations were observed between lipids and prognostic outcome among breast cancer patients overall, or among patients with luminal A and luminal B subtypes. Among HER2-enriched patients, pre-diagnostic triglyceride level was inversely associated with overall mortality.

**Conclusion:**

Our study suggests that pre-diagnostic triglycerides and the HDL-cholesterol/total-cholesterol ratio may independently provide unique information regarding prognostic outcome among triple negative breast cancer patients. However, a small sample size underlines the need for additional studies.

**Electronic supplementary material:**

The online version of this article (10.1186/s12885-018-4568-2) contains supplementary material, which is available to authorized users.

## Background

Studies investigating the influence of metabolic differences on breast cancer prognosis often show contradictory results, and information on breast cancer heterogeneity/subtypes is not included [1–4]. The identification of distinct breast cancer molecular subtypes has warranted more tailored treatment regimes, and a prolonged survival has been observed for the majority of breast cancer patients, but not for all [5]. Importantly, triple negative breast cancer (TNBC), defined by lack of estrogen receptor (ER), progesterone receptor (PR), and human epidermal growth factor receptor-2 (HER2) expression [6], remains associated with shorter disease-free interval and higher mortality rate [7]. Furthermore, prognostic diversity often exists within each molecular subtype [8], and metabolic heterogeneity is likely to be present in all cancers, including breast cancer [9]. Identifying biologic markers associated with metabolic heterogeneity, breast cancer subtype and prognosis is of importance in order to discover potential targets for treatment and optimize breast cancer outcomes.

Dyslipidemia has been independently linked with breast cancer development [10], but studies are conflicting [11]. Moreover, dyslipidemia is strongly associated with obesity. Recently, patients` body mass index (BMI) at diagnosis was observed to be differently distributed across breast cancer molecular subtypes, and obese women were more likely to have TNBC [12], also supported by others [13, 14]. Of note, obesity has been linked to increased risk of recurrence and breast cancer specific mortality [15, 16]. However, a survival study newly demonstrated an association between pre-diagnostic obesity and worse outcome only among patients with Luminal A disease [17]. Moreover, previous studies have shown inconsistent results on pre-diagnostic obesity and TNBC prognosis [18, 19], suggesting there may be other metabolic mechanisms driving the carcinogenesis in more aggressive tumors. Triglycerides serve as an independent source for fatty acid oxidation [20], an important process promoting cell proliferation and tumor growth [21], proposing a carcinogenic potential of triglycerides. However, the relationship between pre-diagnostic triglycerides and breast cancer development by molecular subtype remains unclear [22]. Moreover, cholesterol has been suggested to play a role in breast cancer progression [23]. Conversely, high density lipoprotein (HDL)-cholesterol possesses anti-inflammatory properties [24], and has been inversely associated with breast cancer risk [25], and suggestively breast cancer survival [22]. We have previously observed that low HDL-cholesterol may be associated with higher estrogen levels and absolute mammographic density [26–28]. In addition, different lipoprotein subfractions vary by progesterone receptor expression [29], suggesting the influence of HDL-cholesterol on breast cancer prognosis may differ by breast cancer phenotype [30].

Thus, the main aim of the present study was to investigate whether variations in pre-diagnostic lipid levels in a population-based breast cancer cohort independently affect breast cancer recurrence and mortality by molecular subtypes.

## Material and method

### Study design, settings and participants

The present study includes 464 women diagnosed with primary invasive histological verified breast cancer in the period 1980–2014, who participated in the Tromsø Study during 1979–2008 (Tromsø surveys 2–6) [31]. The Tromsø Study is a population-based prospective study aiming to explore risk factors for chronic diseases. Age-eligible women, including total birth cohorts and random samples of the Tromsø population were recruited: A total of 19,947 women participated (77.0% of invited women).

### Assessment of weight, height and serum lipids

Height and weight were measured upon enrollment in the Tromsø Study (study entry = pre-diagnostic), and BMI (kg/m^2^) was calculated. All attendees had non-fasting blood samples drawn at each study entry. The samples were analyzed at the Department of Laboratory Medicine, University Hospital North Norway, Tromsø (ISO-standard accredited laboratory). Serum triglycerides, total-cholesterol, and HDL-cholesterol were analyzed within 10 h by enzymatic, colorimetric methods and commercially available kits (CHOD-PAP for cholesterol, and GPO-PAP for triglycerides; Boehringer Mannheim). Coefficient of variation (CV) 3.3%. [32]. However, in the Tromsø survey 4 (1994–95), HDL-cholesterol was measured after the precipitation of LDL with heparin and manganese chloride (CV 4.2%) [31, 32].

### Case identification and breast tumor characteristics

Participants were linked to the Cancer Registry of Norway using the unique national 11-digit identification number (Statistics Norway), and 656 women in the cohort were identified and diagnosed with primary invasive breast cancer from 1979 through December 31st 2014. To account for the possibility that undiagnosed cancer could influence our results, we excluded those in whom breast cancer developed during the first year after they entered the cohort (n_cases_ = 12). We also excluded those being < 20 years of age at study entry (n_cases_ = 3), and those with missing information on tumor cell proliferation marker, Ki67 hotspot index (n_cases_ = 123), ER, PR, or HER2 status (n_cases_ = 54). Hence, a total of 464 women with histologically verified invasive primary breast cancer were included.

All tumor samples were fixed in 4% buffered formaldehyde before processing and embedding in paraffin. In order to obtain comparable results due to changes in identifying tumor characteristics over time (1980–2012), and to obtain complete information on tumor characteristics, a majority (n_cases_ = 375) of the tissue samples were analyzed on tissue microarrays (TMA) (Centre for Cancer Biomarkers CCBIO, Section for Pathology, University of Bergen). Immunohistochemistry (IHC) and HER2 Silver in Situ Hybridization (SISH) were employed in TMA, to obtain Ki67 and HER2 status, respectively. HER2-SISH was performed on IHC 2+ cases, and considered positive if HER2/Chr17 ratio by SISH was > 2.0. Tumors were stained for ER and PR, and considered positive if ≥10% of tumor nuclei stained positive [33]. Breast tumors diagnosed after 2012 (n_cases_ = 89) were analyzed using immunohistochemistry for hormone receptor status and Ki67, and immunohistochemistry and fluorescence in situ hybridization for HER2.

We classified the breast tumors into molecular subtypes (13th St Gallen International Breast Cancer Expert Panel) [34] as follows: Luminal A - ER positive, PR positive, HER2 negative, and Ki-67 < 20%; Luminal B - ER positive and/or PR positive, HER2 positive (or HER2 negative and Ki-67 ≥ 20% or PR negative); HER2-enriched - ER negative, PR negative, and HER2 positive; and TNBC - ER negative, PR negative and HER2 negative.

### Outcomes: Mortality and recurrence

Person-time of follow-up was calculated from the date of breast cancer diagnosis until date of recurrence, death, time of emigration, or end of follow-up (December 31st, 2014), whichever event occurred first. We obtained information on death and emigration through linkage to the Norwegian Cause of Death Registry and Statistics Norway. The mean interval between the pre-diagnostic measurements and diagnosis was 18.6 years (standard deviation (SD) 9.23, range 1.01–35.2 years).

We used the following outcomes: 1) overall mortality and overall survival: death of any cause, and the time interval from date of diagnosis to death of any cause, respectively (all breast cancer stages included, stages 1–4); 2) breast cancer-free interval: the time interval from date of diagnosis to breast cancer recurrence or breast cancer mortality (stages 1–3 breast cancer at diagnosis).

### Treatment data

Each patient’s medical chart was reviewed. Details of all treatment regimens were obtained, including type of surgery, chemotherapy, radiotherapy, and endocrine treatment. Date and site of recurrence was collected, and recurrence was defined by local, regional, and/or visceral relapse.

### Statistical methods

We used multivariable Cox proportional hazards regression models to study the association of pre-diagnostic lipid levels on overall mortality and breast cancer-free interval by molecular subtypes. In order to study the association between lipids and outcomes (overall mortality and breast cancer-free interval) among patients with HER2 positive (+) breast cancer, we combined the patients with HER2+ expression from Luminal B subtype and the patients from HER2-enriched subtype.

We categorized lipids into the following groups by tertiles: triglycerides (≤0.82, 0.83–1.22, and ≥ 1.23 mmol/l), total-cholesterol (≤5.14, 5.15–6.25, and ≥ 6.26 mmol/l), and HDL-cholesterol/total-cholesterol ratio (≤0.27, 0.28–0.34, and ≥ 0.35). The triglyceride/HDL-cholesterol ratio (tertile splits: ≤0.47, 0.48–0.75, and ≥ 0.76) was used as a surrogate marker for insulin resistance [35].

Based on our previous results [36], and in order to evaluate our present findings, a total of 57 breast cancer cases were checked for agreement between molecular subtyping based on immunohistochemistry and TMA. We observed an agreement between these two methods in 73% of the breast cancer cases (kappa value 0.63).

Based on suggested biological mechanisms influencing serum lipid levels, breast cancer recurrence and overall and breast cancer specific mortality, several variables were included in the Cox proportional hazard model as potential confounders. Age (continuous), BMI (continuous), and smoking habits (categorical) at blood sampling, age at diagnosis (continuous) and disease stage (categorical) were included as covariates in the final models. To account for secular trends in treatment [http://www.nbcg.no], we adjusted for year of diagnosis, chemotherapy (yes/no) and endocrine treatment (yes/no), but our observations were not significantly influenced. In addition, in order to account for comorbid disease confounding, we adjusted for alcohol habits, physical activity, blood glucose, blood pressure, and time since last meal. Moreover, we excluded women who died within the first and third year after being diagnosed breast cancer to account for serious comorbidity at diagnosis. However, none of these adjustments influenced our results, and they were not included in the final models. In our study including 464 breast cancer patients with129 events we have 80% power to detect a hazard ratio of 1.28 per standard deviation increase in a continuous variable, and 80% power to detect a hazard ratio of 1.63 for an equally distributed binary variable. Consequently, including 40 events, the respective hazard ratios are 1.56 and 2.43.

To test for interaction between lipids and breast cancer molecular subtypes, two-way cross product terms were added to logistic regression and cox regression models. In both logistic and cox regression models, we observed statistically significant interaction between triglycerides and TNBC (*p = 0.042* and *p = 0.025*, respectively), and between the HDL-cholesterol/total-cholesterol ratio and TNBC (*p = 0.033* and *p = 0.065*, respectively) (data not shown in table). The proportional hazards assumption was assessed by visual inspection of log-log survival functions of levels of pre-diagnostic lipids. Among TNBC, log rank-tests were performed to compare differences in survival curves between the tertiles of triglycerides (*p < 0.001*). All statistical tests were two-sided using a significance level of *p* < 0.05, and analysis was conducted using SPSS version 21.0.

In order to increase the sample size to n_cases_ = 641, we used multiple imputation and imputed values in 20 datasets on the following variables: Ki-67, ER-, PR-, and HER2 status, age, disease stage, current smoking, BMI, triglycerides, HDL/total-cholesterol ratio, and total-cholesterol. Separate Cox regression analyses showed results that were not substantially different from what we observed in our complete case analyses, and these data are not presented in the text (TNBC patients presented in Additional file 1: Table S1).

## Results

### Characteristics of the study population

Of the 464 women diagnosed with invasive histologically verified breast cancer (mean age at diagnosis of 57.9 years), a total of 129 died during a mean follow-up of 8.4 years: 51.9% were attributable to breast cancer, 7.9% to other cancers, 7.1% to cardiovascular disease, 16.4% to other causes, and 16.7% to unregistered cause of death. Molecular subtypes were distributed as follows: Luminal A (49.1%), Luminal B (21.3%), HER2-enriched (9.3%) and TNBC (20.3%). Patients diagnosed with the most common subtype, Luminal A, in comparison to patients diagnosed with TNBC, were older at diagnosis (58.8 years vs. 55.9 years, *p = 0.035*), had smaller tumors (21.6 mm vs. 29.2 mm, *p = 0.001*), lower Ki-67 (8.43% vs. 30.5%, *p < 0.001*), and lower overall mortality (19.7% vs. 40.4%, *p < 0.001*) (Table 1). Characteristics of the 123 women excluded due to missing information on Ki-67 did not substantially differ from the final study population (data not shown).

**Table 1:** Descriptive characteristics among breast cancer patients by molecular subtypes

Molecular subtypes	All cases (*N = 464*)^a^	Luminal A (*N = 228*)^a^	Luminal B (*N = 99*)^a^	HER-2 enriched (*N = 43*)^a^	TNBC (*N = 94*)^a^
	Mean (SD) / %	Mean (SD) / %	Mean (SD) / %	Mean (SD) / %	Mean (SD) / %
Characteristics at study entry					
Age at blood sampling, years	39.5 (14.5)	40.3 (15.2)	36.5 (10.7)	41.2 (15.1)	39.9 (14.7)
Age at diagnosis, years	57.9 (12.6)	58.8 (12.8)	57.0 (10.3)	59.0 (13.3)	55.9 (13.8)
Follow-up after diagnosis, years	8.43 (6.93)	9.35 (6.78)	6.43 (5.59)	7.27 (6.83)	8.72 (8.23)
Clinical variables^b^					
SBP, mmHg	125 (19.4)	125 (19.5)	123 (15.9)	129 (24.5)	126 (20.3)
Waist-hip ratio	0.83 (0.07)	0.84 (0.08)	0.81 (0.06)	0.82 (0.05)	0.84 (0.07)
BMI, kg/m^2^	23.4 (3.68)	23.3 (3.26)	23.3 (3.50)	23.3 (3.86)	24.1 (4.80)
Reproductive factors^b^					
Number of children, number	1.93 (1.39)	1.88 (1.33)	1.63 (1.11)	2.40 (1.96)	2.26 (1.53)
Age at menarche, years	13.3 (1.39)	13.2 (1.50)	13.4 (1.28)	13.2 (1.09)	13.4 (1.30)
HRT use, %	30.5	31.5	28.7	28.6	30.5
Serum samples^b^					
Total cholesterol, mmol/l	5.82 (1.30)	5.76 (1.22)	5.77 (1.30)	6.03 (1.32)	5.95 (1.46)
HDL-cholesterol, mmol/l	1.75 (0.40)	1.76 (0.40)	1.72 (0.32)	1.67 (0.38)	1.78 (0.45)
Triglycerides, mmol/l	1.16 (0.76)	1.11 (0.59)	1.12 (0.53)	1.35 (1.23)	1.28 (1.14)
Lifestyle factors^b^					
Moderate physical activity, %	72.5	72.8	75.3	72.7	68.2
Current smokers, %	40.4	40.4	38.1	48.5	40.0
Tumor characteristics					
Tumor size, mm	24.6 (19.5)	21.6 (18.6)	26.1 (17.8)	30.3 (19.1)	29.2 (23.0)
Number of metastatic lymph nodes	2.03 (3.92)	1.46 (3.23)	3.04 (4.66)	2.00 (2.97)	2.43 (4.67)
Stage, %	1.77 (0.81)	1.59 (0.68)	1.92 (0.80)	2.12 (0.96)	1.95 (0.93)
1	41.7	51.3	31.6	27.9	35.5
2	39.6	38.9	49.0	30.2	35.5
3	15.7	8.80	15.3	37.2	22.9
4	3.00	0.90	4.10	4.70	6.50
Histologic grading, %	1.92 (0.73)	1.55 (0.57)	2.13 (0.68)	2.54 (0.51)	2.49 (0.65)
1	30.1	48.6	17.8	2.60	8.60
2	45.8	47.6	53.3	42.1	34.6
3	24.1	3.80	28.9	55.3	56.8
Estrogen positive, %	64.2	91.2	88.7	0	0
Progesterone positive, %	47.9	71.6	61.3	0	0
HER2 positive, %	16.0	0	34.0	100	0
Ki-67%	18.9 (17.3)	8.43 (4.98)	30.4 (16.1)	27.9 (17.1)	30.5 (22.0)
Treatment					
Type of surgery					
Breast conserving surgery	45.6	54.5	45.1	33.0	27.0
Mastectomy	52.5	44.6	51.6	66.7	70.3
Others^c^	1.90	0.90	3.30	0.30	2.70
Chemotherapy, %	38.2	25.9	52.6	51.5	49.4
Radiation therapy, %	48.6	66.8	67.0	39.4	54.1
Endocrine therapy, %	50.3	55.7	70.1	21.2	24.7
Outcome					
Recurrence, %	17.0	13.2	17.2	30.2	21.0
Overall mortality, %	27.8	19.7	29.3	39.5	40.4
Breast cancer mortality, %	14.4	5.27	20.2	27.9	24.5

^a^Numbers may vary due to missing information

^b^Clinical variables, reproductive factors, serum samples, and lifestyle factors at study entry

^c^Other types of surgery include primary reconstruction and oncoplastic surgery

Abbreviations: *BMI* body mass index, *HDL* high density lipoprotein, *HRT* hormone replacement therapy, *IDC* invasive ductal carcinoma, *ILC* invasive lobular carcinoma, *n* number of cases, *SPB* systolic blood pressure

### 5-year overall survival and 5-year breast cancer-free interval by molecular subtypes

The 5-year overall survival for the entire cohort was 83% (data not shown in tables or figures). When stratified by tumor subtypes, women with Luminal A breast cancer had a 5-year overall survival of 89%, and a 5-year breast cancer-free interval of 92%. Those with HER2-enriched and TNBC subtypes had 5-year overall survival of 73 and 75%, respectively, and 5-year breast cancer-free interval of 74 and 84%, respectively (Additional file 2: Figure S1 and Additional file 3: Figure S2).

### Pre-diagnostic lipids and total mortality

Table 2 presents the multivariable adjusted hazard ratios (HRs) for all-cause mortality in relation to pre-diagnostic lipids and breast cancer molecular subtypes. No association was observed between triglycerides, total-cholesterol, the HDL-cholesterol/total-cholesterol ratio, and the triglyceride/HDL-cholesterol ratio and overall mortality among all breast cancer patients combined, or among Luminal A and B subtypes.

**Table 2:** Multivariable adjusted hazard ratios for overall mortality by pre-diagnostic lipids and breast cancer molecular subtypes

	All cases (events^a^=129)		Luminal A (events^a^=45)		Luminal B (events^a^=29)		HER2-enriched (events^a^=17)		TNBC (events^a^=38)	
	n	HR (95% CI)	n	HR (95% CI)	n	HR (95% CI)	n	HR (95% CI)	n	HR (95% CI)
Triglycerides										
Continuous, mmol/l	464	1.01 (0.88–1.22)	228	0.99 (0.56–1.75)	99	1.64 (0.78–3.46)	43	0.06 (0.01–0.77)	94	1.02 (0.83–1.24)
Tertiles										
≤ 0.82 mmol/l	153	1.00	77	1.00	33	1.00	14	1.00	29	1.00
0.83–1.22 mmol/l	158	0.69 (0.42–1.11)	78	1.19 (0.53–2.66)	33	0.50 (0.17–1.46)	14	0.13 (0.03–0.64)	33	0.96 (0.35–2.62)
≥ 1.23 mmol/l	153	1.24 (0.78–1.99)	73	1.26 (0.57–2.78)	33	1.01 (0.36–2.78)	15	0.06 (0.01–0.55)	32	2.99 (1.17–7.63)
p-trend		*0.351*		*0.838*		*0.179*		*0.001*		*0.011*
Total-cholesterol										
Continuous, mmol/l	464	1.03 (0.90–1.19)	228	1.06 (0.83–1.36)	99	1.00 (0.66–1.51)	43	1.05 (0.70–1.57)	94	1.05 (0.77–1.44)
Tertiles										
≤ 5.14 mmol/l	155	1.00	77	1.00	37	1.00	11	1.00	30	1.00
5.15–6.25 mmol/l	156	1.33 (0.80–2.22)	82	1.66 (0.70–3.95)	27	0.85 (0.29–2.50)	16	1.28 (0.18–7.93)	31	1.01 (0.39–2.67)
≥ 6.26 mmol/l	153	1.46 (0.87–2.44)	69	1.48 (0.61–3.60)	35	1.20 (0.38–3.72)	16	2.04 (0.34–12.3)	33	1.41 (0.49–4.06)
p-trend		*0.354*		*0.512*		*0.830*		*0.533*		*0.731*
HDL-cholesterol/total-cholesterol ratio										
Continuous,	464	0.89 (0.58–1.36)	228	0.68 (0.02–25.0)	99	0.23 (0.01–54.2)	43	693 (0.06–747)	94	0.02 (0.00–1.39)
Tertiles										
≤ 0.27	154	1.00	76	1.00	32	1.00	21	1.00	25	1.00
0.28–0.34	154	0.84 (0.65–1.29)	67	0.81 (0.37–1.79)	37	1.48 (0.57–3.87)	14	1.53 (0.45–5.23)	36	0.53 (0.24–1.19)
≥ 0.35	156	0.82 (0.51–1.33)	85	1.05 (0.43–2.00)	30	0.96 (0.33–2.74)	8	5.61 (0.62–21.0)	33	0.33 (0.12–0.89)
p-trend		*0.385*		*0.819*		*0.986*		*0.176*		*0.026*

Multivariable Cox proportional hazard regression models

^a^Number of deaths

Adjusted for age (continuous), Body mass index (continuous), and current smoking (categorical) at blood sampling, age at diagnosis (continuous), and disease stage (categorical)

Abbreviations: *HDL* high density lipoprotein, *HR* hazard ratio; *n* number of cases, *t* Total, *TNBC* triple negative breast cancer

**Fig. 1:**
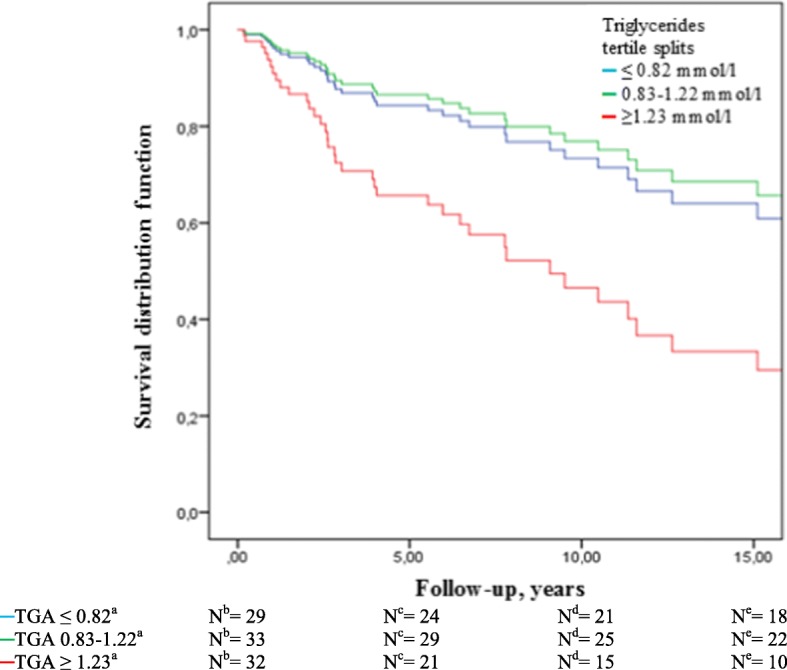
Overall survival among triple negative breast cancer (TNBC) patients by tertiles of triglycerides. ^a^mmol/l, ^b^Number of cases at diagnosis, ^c^Number of cases in follow-up after 5 years, ^d^Number of cases in follow-up after 10 years, ^e^Number of cases in follow-up after 15 years. Abbreviations: *N*, number of cases; TGA, triglycerides

Compared to patients with TNBC in the lowest tertile of triglycerides, those in the highest tertile (≥ 1.23 mmol/l) had a 3 times higher overall mortality (HR 2.99, 95% CI 1.17–7.63) (Table 2) and 19% lower 5-year overall survival (65% vs. 84%) (Fig. 1). TNBC patients in the highest (≥0.35) vs. lowest (0.27) tertile of the HDL/total-cholesterol ratio, had a 67% reduced overall mortality risk (HR 0.33, 95% CI 0.12–0.89) (Table 2).

Among women with HER2+ breast cancer, those in the highest vs. lowest tertile of triglycerides had an 86% reduced overall mortality risk (HR 0.14, 95% CI 0.03–0.60, *p-trend 0.038*) (Additional file 4: Table S2).

The triglyceride/HDL-cholesterol ratio was positively associated with overall mortality among TNBC patients (Additional file 5: Table S3).

### Pre-diagnostic lipids and breast cancer-free interval

Table 3 presents the multivariable HRs for breast cancer-free interval by pre-diagnostic lipids and molecular subtypes. We observed no association between lipid levels and breast cancer-free interval among all breast cancer patients combined, or among Luminal A, Luminal B, or HER2-enriched molecular subtypes.

**Table 3:** Multivariable adjusted hazard ratios for breast cancer-free interval^a^ by pre-diagnostic lipids and molecular subtypes

	All cases (events^b^=91)		Luminal A (events^b^=31)		Luminal B (events^b^=21)		HER2-enriched (events^b^=15)		TNBC (events^b^=24)	
	N	HR (95% CI)	n	HR (95% CI)	n	HR (95% CI)	n	HR (95% CI)	n	HR (95% CI)
Triglycerides										
Continuous, mmol/l	446	1.01 (0.88–1.22)	224	0.88 (0.40–1.92)	94	0.76 (0.23–2.46)	41	1.01 (0.71–1.47)	87	1.12 (0.73–1.64)
Tertiles										
≤ 0.82 mmol/l	148	1.00	76	1.00	33	1.00	13	1.00	26	1.00
0.83–1.22 mmol/l	152	0.81 (0.47–1.40)	77	1.26 (0.51–3.11)	31	0.24 (0.06–0.89)	13	0.27 (0.04–1.91)	31	1.37 (0.37–4.98)
≥ 1.23 mmol/l	146	1.23 (0.71–2.13)	71	1.02 (0.38–2.68)	30	0.42 (0.12–1.52)	15	0.26 (0.03–2.54)	30	5.63 (1.64–19.3)
p-trend		*0.308*		*0.854*		*0.081*		*0.422*		*0.004*
HDL-cholesterol/total-cholesterol ratio										
Continuous	446	1.18 (0.09–15.7)	224	16.4 (0.18–1.4e^4^)	94	15.0 (0.04–6.2e^4^)	41	116 (0.00–4.3e^6^)	87	0.03 (0.00–6.86)
Tertiles										
≤ 0.27	147	1.00	74	1.00	29	1.00	21	1.00	23	1.00
0.28–0.34	149	1.16 (0.70–1.93)	66	1.52 (0.58–4.02)	36	5.13 (0.98–20.7)	13	1.27 (0.31–5.21)	34	0.58 (0.22–1.56)
≥ 0.35	150	1.02 (0.58–1.78)	84	1.87 (0.69–5.07)	29	2.06 (0.55–7.75)	7	2.99 (0.43–20.8)	30	0.32 (0.10–1.06)
p-trend		*0.808*		*0.219*		*0.344*		*0.310*		*0.060*

Multivariable Cox proportional hazard regression models

^a^Breast cancer-free interval among women staged 1–3 at diagnosis

^b^Number of breast cancer patients with recurrence or death from breast cancer

Adjusted for age (continuous), Body mass index (continuous), and current smoking (categorical) at blood sampling, age at diagnosis (continuous), and disease stage (categorical)

Abbreviations: *HDL* high density lipoprotein, *HR* hazard ratio, *n* number of cases, *TNBC* triple negative breast cancer

**Fig. 2:**
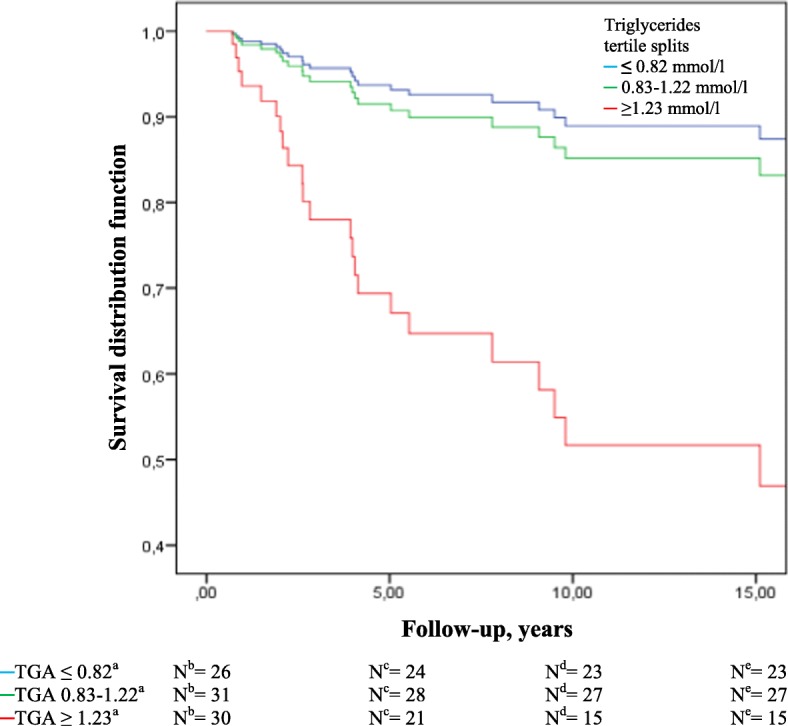
Breast cancer-free interval among triple negative breast cancer (TNBC) patients by tertiles of triglycerides. ^a^mmol/l, ^b^Number of cases at diagnosis, ^c^Number of breast cancer-free cases in follow-up after 5 years, ^d^Number of breast cancer-free cases in follow-up after 10 years, ^e^Number of breast cancer-free cases in follow-up after 15 years. Abbreviations: *N*, number of cases; TGA, triglycerides

Among TNBC, women in the highest (≥1.23 mmol/l) vs. lowest (≤0.82 mmol/l) tertile of triglycerides had 5.6 times higher risk for recurrence or death from breast cancer (HR 5.63, 95% CI 1.64–19.3) (Table 3), and the 5-year breast cancer-free interval was 24% lower for women in the highest vs. lowest tertile of triglycerides (69% vs. 93%) (Fig. 2).

No associations were observed between triglycerides and breast cancer-free interval among HER2+ patients (Additional file 5: Table S3), or between total-cholesterol and breast cancer-free interval overall or by molecular subtypes (data not presented in table).

## Discussion

We observed strong associations between pre-diagnostic triglycerides and overall mortality and breast cancer-free interval among TNBC patients, and the 5-year overall survival was 19% lower for patients in the highest tertile of triglycerides compared to those in the lowest tertile of triglycerides (65% vs. 84%). Women with TNBC and in the highest tertile of triglycerides had 24% lower 5-year breast cancer-free interval (69% vs. 93%) compared to those in the lowest tertile. Conversely, among patients with HER2+ disease, a high serum level of triglycerides was inversely associated with overall mortality. Additionally, the HDL-cholesterol/total-cholesterol ratio was inversely associated with overall mortality among TNBC patients.

Observations in the present study extend previous observations linking variation in pre-diagnostic serum lipids, and in particular triglycerides, to breast cancer prognosis by molecular subtypes. Recently, a Chinese retrospective case series including 221 lean (BMI < 25 kg/m^2^) TNBC patients observed that high triglyceride/HDL-cholesterol ratio, but not triglyceride, was associated with poor overall survival [37]. In another case series, an inverse association was observed between HDL-cholesterol at diagnosis of breast cancer and recurrence and overall mortality among 394 TNBC patients, but there was no association between triglycerides and breast cancer outcomes [30]. Another Chinese study including 1044 breast cancer patients, unclassified by molecular subtype, observed that preoperative lower triglycerides and HDL-cholesterol were associated with shorter disease-free and worse overall survival, respectively [22]. In contrast to our study, triglycerides and HDL-cholesterol were measured at diagnosis in these studies, and one may argue that lipid metabolism could have been altered by metabolic changes due to the breast cancer disease [38].

In the present study, we observed a protective effect of the HDL-cholesterol/total-cholesterol ratio on overall mortality only among the TNBC patients. We have recently observed that low HDL-cholesterol is associated with more aggressive tumor characteristics [29]. A low level of HDL-cholesterol has been inversely associated with the activity of the pro-inflammatory cytokine interleukin (IL)-6 [39]. Moreover, IL-6 and IL-8 may promote tumor progression in TNBC cells [40]. Interestingly, it has been proposed that HDL-cholesterol possess anti-tumorigenic properties through regulation of angiogenesis, involving lowered expression of vascular endothelial growth factor (VEGF) [41]. Studies show that high expression of VEGF correlates with metastatic potential of TNBC [42], suggesting a potential biological link between low HDL-cholesterol and tumor progression among these patients. There are plausible biologic mechanisms supporting a role of triglycerides in tumor proliferation, growth and metastasis among patients with TNBC: Triglycerides play a role in energy storage, and they serve as a source for fatty acid oxidation (FAO), an important energy source for cell proliferation and migration. Overexpressed FAO is associated with more aggressive tumors [43], and studies show that metastatic TNBC cell lines maintain high levels of ATP through up-regulated fatty acid oxidation [44]. Triglyceride/HDL ratio may serve as a surrogate marker of insulin resistance [35], and insulin resistance further stimulates triglyceride production through lipolysis, and de novo fatty acid synthesis in the liver [45]. Both insulin resistance and triglycerides correlate with presence of white adipose tissue (WAT) inflammation in the breast, a proposed link between chronic subclinical inflammation, breast cancer aggressiveness and worse breast cancer prognosis [46]. This correlation remains independent of patients` BMI [47], suggesting WAT inflammation and associated metabolic obesity, including high triglycerides, is a stronger driver of breast cancer progression than obesity alone.

We observed an inverse association between triglycerides and overall mortality among patients overexpressing HER2. This observation is in contrast to some previous observations on HER2+ tumors which suggest a reduced tumor proliferation from the inhibitory effect of polyunsaturated fatty acids on fatty acid synthase [48, 49]. While being inversely associated to serum levels of polyunsaturated fatty acids, triglyceride levels correspond to serum levels of the saturated fatty acid, palmitate [50], and studies show that endogeneous palmitate may be toxic to HER2+ cells [51]. Therefore, a deeper understanding of the biologic mechanisms underlying these results are needed.

Our study has several strengths. Although we can not fully exclude selection and diagnostic bias, the high attendance rate (77%) in the population-based Tromsø study and completeness of identification of breast cancer cases due to mandatory registration of all new cases through the Cancer Registry of Norway (historically 98.8% complete) [52], indicate that the results are highly representative of the source population. Additionally, continuous registration of death (Norwegian Cause of Death Registry) and emigration (Statistics of Norway) limits loss to follow-up and missing endpoint data. Moreover, all medical charts were systematically reviewed with tumor and treatment characteristics abstracted. A majority of the tumors’ histopathology was reanalyzed by tissue microarrays [36], enabling complete tumor characteristics and comparison between invasive breast cancer cases and molecular subtypes diagnosed at various time points. We observed agreement between molecular subtyping based on immunohistochemistry and tissue microarrays, supporting our tissue microarrays reanalyzing. Moreover, our long follow-up (mean 8.4 years) increases the chance of registering late recurrences.

A limitation of our study is the small number of patients in each molecular subclass, and the study may be underpowered to detect the hypothesized differences in all the distinct subclasses, which underlines the need for additional studies. Furthermore, as a result of limited number of events and in accordance with other studies [53, 54], we used breast cancer-free interval instead of breast cancer specific mortality as one of our endpoints. In order to classify all cases into molecular subclasses, a total of 123 women were excluded due to missing information on Ki67. However, characteristics of these women did not substantially differ from the study population. Furthermore, patients were diagnosed during a wide time period (1980–2014) with possible secular treatment effect, but reanalyzing histological classifications and adjusting for year of diagnosis and adjuvant systemic treatment according to national uniform guidelines by the Norwegian Breast Cancer Group [http://www.nbcg.no], did not influence our main results. Comorbidity may be a potential confounder when studying breast cancer survival, and missing information on comorbid disease can be a concern. We used s-glucose, blood pressure, BMI, physical activity, alcohol and smoking habits as markers of comorbid disease. However, additional data on comorbidity may potentially add valuable information to our results [55]. Blood samples were not collected in a fasting state, which can affect lipid levels, but we adjusted for time since last meal in our analyses. Although we adjusted for BMI, we could not adjust for other variables that affect lipid levels such as diet, genetics or familial predisposition, as the data were not available.

## Conclusions

Our study supports an association between pre-diagnostic triglycerides and the HDL-cholesterol/total-cholesterol ratio with survival for TNBC breast cancer patients. High triglycerides may be a negative prognostic marker, while pre-diagnostic high HDL-cholesterol/total-cholesterol ratio suggests improved prognosis. These findings are supported by plausible biological mechanisms linking triglycerides and the HDL-cholesterol/total-cholesterol ratio to breast cancer growth and progression. TNBC is associated with poor prognosis, and identifying and incorporating clinically available biomarkers is of great importance in order to improve the outcomes for this subgroup of breast cancer patients. Additional and larger studies including molecular subtyping, as well as more detailed information on comorbidity, are needed to define the clinical implications of these findings. Our findings may encourage closer follow-up of women at risk and future clinical trials to test effects of lipid-altering medications on breast cancer prognosis.

## Additional files

Additional file 1: **Table S1.** Multivariable adjusted Cox proportional hazard ratios (HRs) for overall mortality (n_cases_ = 114) and breast cancer-free interval (n_cases_ = 107) by pre-diagnostic triglycerides and HDL/total-cholesterol ratio among triple negative breast cancer (TNBC) patients in the imputed data set (DOCX 16 kb)

Additional file 2: **Figure S1.** Age-adjusted overall survival by breast cancer molecular subtypes^a^. ^a^ Luminal A - ER positive, PR positive, HER2 negative, and Ki-67 < 20%; Luminal B - ER positive and/or PR positive, HER2 positive (or HER2 negative and Ki-67 ≥ 20% or PR negative); HER2-enriched - ER negative, PR negative, and HER2 positive; and TNBC - ER negative, PR negative and HER2 negative. Abbreviations: ER, estrogen receptor; HER2, human epidermal growth factor receptor-2; PR, progesterone receptor; TNBC, triple negative breast cancer (PDF 215 kb)

Additional file 3: **Figure S2.** Age-adjusted breast cancer-free interval by breast cancer molecular subtypes^a^. ^a^ Luminal A - ER positive, PR positive, HER2 negative, and Ki-67 < 20%; Luminal B - ER positive and/or PR positive, HER2 positive (or HER2 negative and Ki-67 ≥ 20% or PR negative); HER2-enriched - ER negative, PR negative, and HER2 positive; and TNBC - ER negative, PR negative and HER2 negative. Abbreviations: ER, estrogen receptor; HER2, human epidermal growth factor receptor-2; PR, progesterone receptor; TNBC, triple negative breast cancer (PDF 215 kb)

Additional file 4: **Table S2.** Multivariable adjusted Cox proportional hazard ratios (HRs) for overall mortality and breast cancer-free interval by pre-diagnostic triglycerides among HER2+ patients (DOCX 16 kb)

Additional file 5: **Table S3.** Multivariable adjusted Cox proportional hazard ratios (HRs) for overall mortality and breast cancer-free interval by pre-diagnostic triglycerides/HDL-cholesterol ratio among triple negative breast cancer (TNBC) patients (DOCX 16 kb)

## Abbreviations

BMI: Body mass index
CCBIO: Centre for cancer biomarkers
CI: Confidence interval
CV: Coefficient of variation
EBBA: Energy balance and breast cancer aspect
ER: Estrogen receptor
FAO: Fatty acid oxidation
HDL: High density lipoprotein
HER2: Human epidermal growth factor receptor2
HR: Hazard ratio
IHC: Immunohistochemistry
IL: Interleukin
n: Number
PR: Progesterone receptor
SISH: Silver in situ hybridization
TMA: Tissue microarray
VEGF: Vascular endothelial growth factor
WAT: White adipose tissue
