# Supplementary material for: Impact of pre-diagnostic triglycerides and HDL-cholesterol on breast cancer recurrence and survival by breast cancer subtypes
Source: BMC Cancer. 2018 Jun 15;18:654. doi: 10.1186/s12885-018-4568-2 (PMC6003110; doi:10.1186/s12885-018-4568-2)
Supplement: Supplementary file 1 — Table S1. Multivariable adjusted Cox proportional hazard ratios (HRs) for overall mortality (ncases = 114) and breast cancer-free interval (ncases = 107) by pre-diagnostic triglycerides and HDL/total-cholesterol ratio among triple negative breast cancer (TNBC) patients in the imputed data set (DOCX 16 kb) [file 12885_2018_4568_MOESM1_ESM.docx]

| **Table S1.** Multivariable adjusted Cox proportional hazard ratios (HRs) for overall mortality (n_cases_=114) and breast cancer-free interval (n_cases_=107) by pre-diagnostic triglycerides and HDL/total-cholesterol ratio among triple negative breast cancer (TNBC) patients in the imputed data set. | | |  |
| --- | --- | --- | --- |
|  | **Overall mortality** | **Breast cancer-free interval** |  |
|  | HR (95% CI) | HR (95% CI) |  |
| **Triglycerides** |  |  |  |
| *Continuous,* mmol/l | 1.00 (0.82-1.22) | 1.07 (0.73-1.57) |  |
|  |  |  |  |
| *Tertiles* |  |  |  |
| ≤ 0.82 mmol/l | 1.00 | 1.00 |  |
| 0.83 – 1.22 mmol/l | 0.63 (0.23-1.69) | 0.76 (0.25-2.30) |  |
| ≥ 1.23 mmol/l | 2.02 (0.79-5.18) | 3.02 (1.10-8.30) |  |
| *P-trend* | *0.081* | *0.024* |  |
|  |  |  |  |
| **HDL-cholesterol/total-cholesterol ratio** |  |  |  |
| *Continuous* | 0.04 (0.00-3.90) | 0.05 (0.00-6.54) |  |
|  |  |  |  |
| *Tertiles* |  |  |  |
| ≤ 0.27 | 1.00 | 1.00 |  |
| 0.28 – 0.34 | 0.57 (0.25-1.30) | 0.58 (0.23-1.43) |  |
| ≥ 0.35 | 0.32 (0.12-0.89) | 0.38 (0.13-1.08) |  |
| *P-trend* | *0.024* | *0.064* |  |
|  |  |  |  |
| Multivariable Cox proportional hazard regression models.  Adjusted for age (continuous), body mass index (continuous), and current smoking (categorical) at blood sampling, age at diagnosis (continuous), and disease stage (categorical).  Abbreviations: CI, confidence interval; HDL, high density lipoprotein | | |  |
