# Supplementary material for: Impact of pre-diagnostic triglycerides and HDL-cholesterol on breast cancer recurrence and survival by breast cancer subtypes
Source: BMC Cancer. 2018 Jun 15;18:654. doi: 10.1186/s12885-018-4568-2 (PMC6003110; doi:10.1186/s12885-018-4568-2)
Supplement: Supplementary file 3 — Figure S2. Age-adjusted breast cancer-free interval by breast cancer molecular subtypesa. a Luminal A - ER positive, PR positive, HER2 negative, and Ki-67 < 20%; Luminal B - ER positive and/or PR positive, HER2 positive (or HER2 negative and Ki-67 ≥ 20% or PR negative); HER2-enriched - ER negative, PR negative, and HER2 positive; and TNBC - ER negative, PR negative and HER2 negative. Abbreviations: ER, estrogen receptor; HER2, human epidermal growth factor receptor-2; PR, progesterone receptor; TNBC, triple negative breast cancer (PDF 215 kb) [file 12885_2018_4568_MOESM3_ESM.pdf]

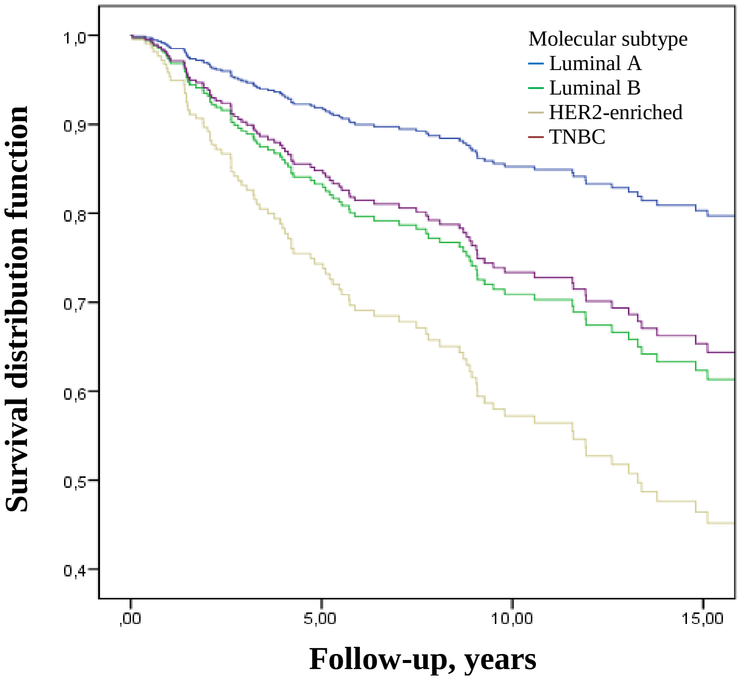

Supplementary figure 2 Age-adjusted breast cancer-free interval by breast cancer molecular subtypes[a]

[a] Luminal A - ER positive, PR positive, HER2 negative, and Ki-67 <20%; Luminal B - ER positive and/or PR positive, HER2 positive (or HER2 negative and Ki-67≥20% or PR negative); HER2-enriched - ER negative, PR negative, and HER2 positive; and TNBC - ER negative, PR negative and HER2 negative.

Abbreviations: ER, estrogen receptor; HER2, human epidermal growth factor receptor-2; PR, progesterone receptor; TNBC, triple negative breast cancer.
