# Supplementary material for: Impact of pre-diagnostic triglycerides and HDL-cholesterol on breast cancer recurrence and survival by breast cancer subtypes
Source: BMC Cancer. 2018 Jun 15;18:654. doi: 10.1186/s12885-018-4568-2 (PMC6003110; doi:10.1186/s12885-018-4568-2)
Supplement: Supplementary file 4 — Table S2. Multivariable adjusted Cox proportional hazard ratios (HRs) for overall mortality and breast cancer-free interval by pre-diagnostic triglycerides among HER2+ patients (DOCX 16 kb) [file 12885_2018_4568_MOESM4_ESM.docx]

| **Table S2.** Multivariable adjusted Cox proportional hazard ratios (HRs) for overall mortality and breast cancer-free interval by pre-diagnostic triglycerides among HER2+ patients. | | | | |  |
| --- | --- | --- | --- | --- | --- |
|  |  | **Overall mortality**  (events^a^= 28) | **Breast cancer-free interval**  (events^b^= 22) | |  |
|  | *N* ^c^ | HR (95% CI) | *N* ^d^ | HR (95% CI) |  |
| **Triglycerides** |  |  |  | |  |
| *Continuous* | 72 | 0.49 (0.12-1.93) | 71 | 1.12 (0.78-1.57) |  |
|  |  |  |  | |  |
| *Tertiles* |  |  |  | |  |
| ≤ 0.82 mmol/l | 27 | 1.00 | 26 | 1.00 |  |
| 0.83 – 1.22mmol/l | 19 | 0.34 (0.11-0.99) | 19 | 0.77 (0.24-2.49) |  |
| ≥ 1.23 mmol/l | 26 | 0.14 (0.03-0.60) | 26 | 0.47 (0.11-2.09) |  |
| *P-trend* |  | *0.038* |  | *0.328* |  |
|  |  |  |  | |  |
| Multivariable Cox proportional hazard regression models.  ^a^ Number of deaths.  ^b^ Number of breast cancer recurrence or death from breast cancer.  ^c^ Number of patients with HER2+ disease, stages 1-4 at diagnosis  ^d^ Number of patients with HER2+ disease, stages 1-3 at diagnosis  Adjusted for age (continuous), body mass index (continuous), and current smoking (categorical) at blood sampling, age at diagnosis (continuous), and disease stage (categorical)  Abbreviations: CI, confidence interval; HER2, human epidermal growth factor receptor-2 | | | | |  |
