# Supplementary material for: Impact of pre-diagnostic triglycerides and HDL-cholesterol on breast cancer recurrence and survival by breast cancer subtypes
Source: BMC Cancer. 2018 Jun 15;18:654. doi: 10.1186/s12885-018-4568-2 (PMC6003110; doi:10.1186/s12885-018-4568-2)
Supplement: Supplementary file 5 — Table S3. Multivariable adjusted Cox proportional hazard ratios (HRs) for overall mortality and breast cancer-free interval by pre-diagnostic triglycerides/HDL-cholesterol ratio among triple negative breast cancer (TNBC) patients (DOCX 16 kb) [file 12885_2018_4568_MOESM5_ESM.docx]

| **Table S3.** Multivariable adjusted Cox proportional hazard ratios (HRs) for overall mortality and breast cancer-free interval by pre-diagnostic triglycerides/HDL-cholesterol ratio among triple negative breast cancer (TNBC) patients. | | | | |  |
| --- | --- | --- | --- | --- | --- |
|  |  | **Overall mortality**  (events^a^= 38) | **Breast cancer-free interval**  (events^b^= 24) | |  |
|  | *N* ^c^ | HR (95% CI) | *N* ^d^ | HR (95% CI) |  |
| **Triglycerides/HDL-cholesterol ratio** |  |  |  | |  |
| *Continuous* | 94 | 1.00 (0.89-1.12) | 87 | 0.98 (0.68-1.42) |  |
|  |  |  |  | |  |
| *Tertiles* |  |  |  | |  |
| ≤0.47 | 31 | 1.00 | 29 | 1.00 |  |
| 0.48-0.75 | 33 | 1.13 (0.40-3.15) | 31 | 1.81 (0.57-5.74) |  |
| ≥076. | 30 | 3.87 (1.41-10.6) | 27 | 3.66 (1.05-12.7) |  |
| *P-trend* |  | *0.005* |  | *0.038* |  |
|  |  |  |  | |  |
| Multivariable Cox proportional hazard regression models.  ^a^ Number of deaths.  ^b^ Number of breast cancer recurrence or death from breast cancer  ^c^ Number of patients with TNBC, stages 1-4 at diagnosis  ^d^ Number of patients with TNBC, stages 1-3 at diagnosis  Adjusted for age (continuous), body mass index (continuous), and current smoking (categorical) at blood sampling, age at diagnosis (continuous), and disease stage (categorical)  Abbreviations: CI, confidence interval | | | | |  |
